# Supplementary figures and images for: Overexpressing GmCGS2 Improves Total Amino Acid and Protein Content in Soybean Seed
Source: Int J Mol Sci. 2023 Sep 15;24(18):14125. doi: 10.3390/ijms241814125 (PMC10532240; doi:10.3390/ijms241814125)

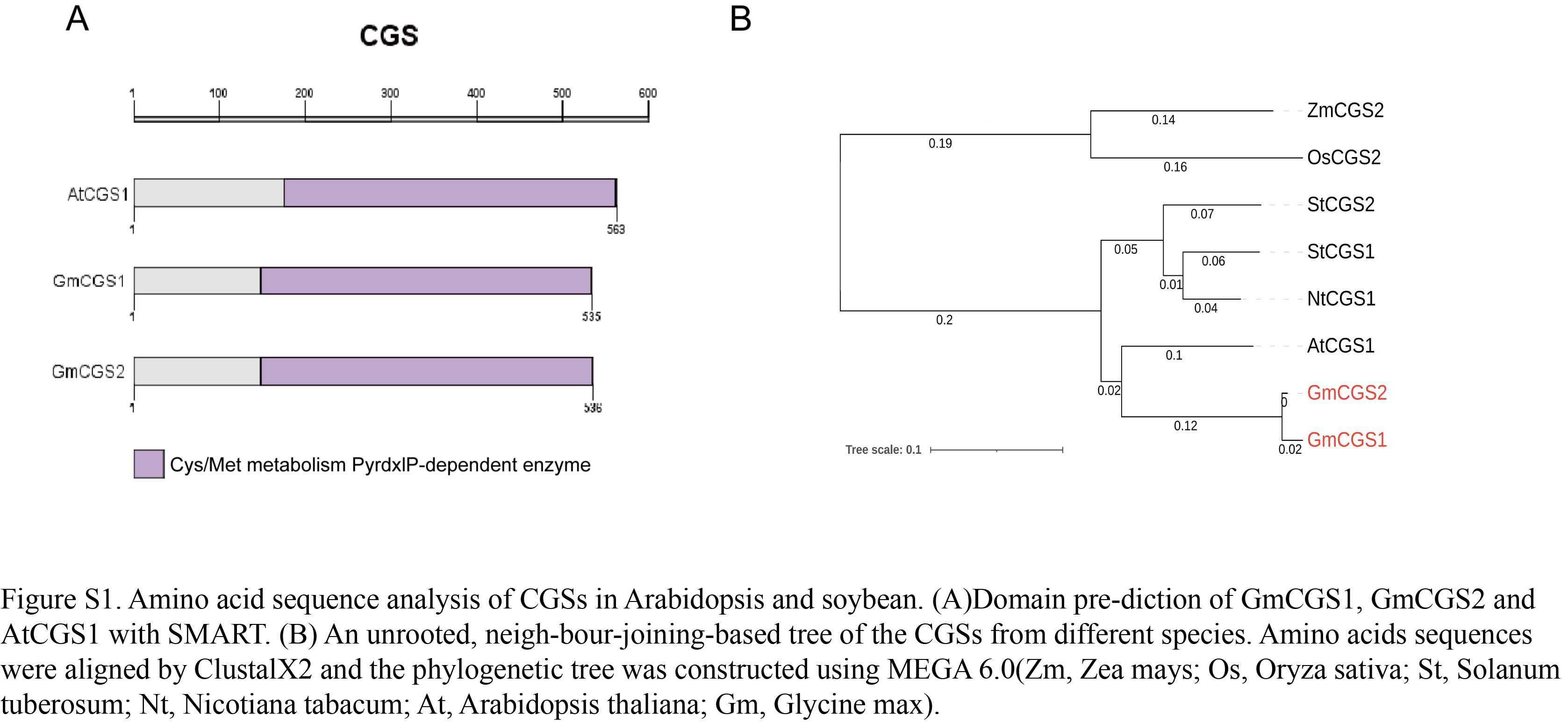

Supplement: Supplementary file 1 [file ijms-24-14125-s001.zip › Figure S1.jpg]

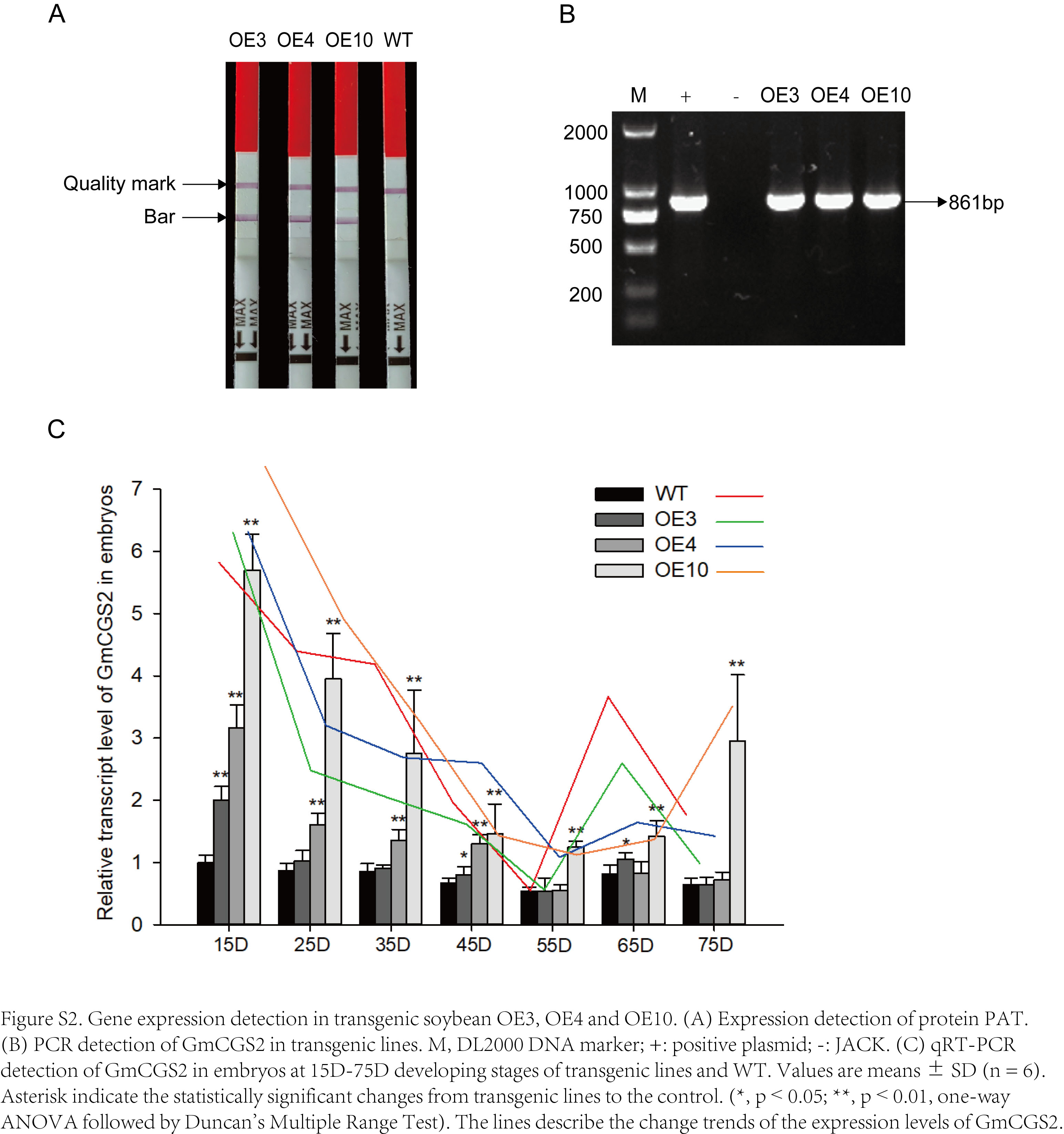

Supplement: Supplementary file 1 [file ijms-24-14125-s001.zip › Figure S2.jpg]

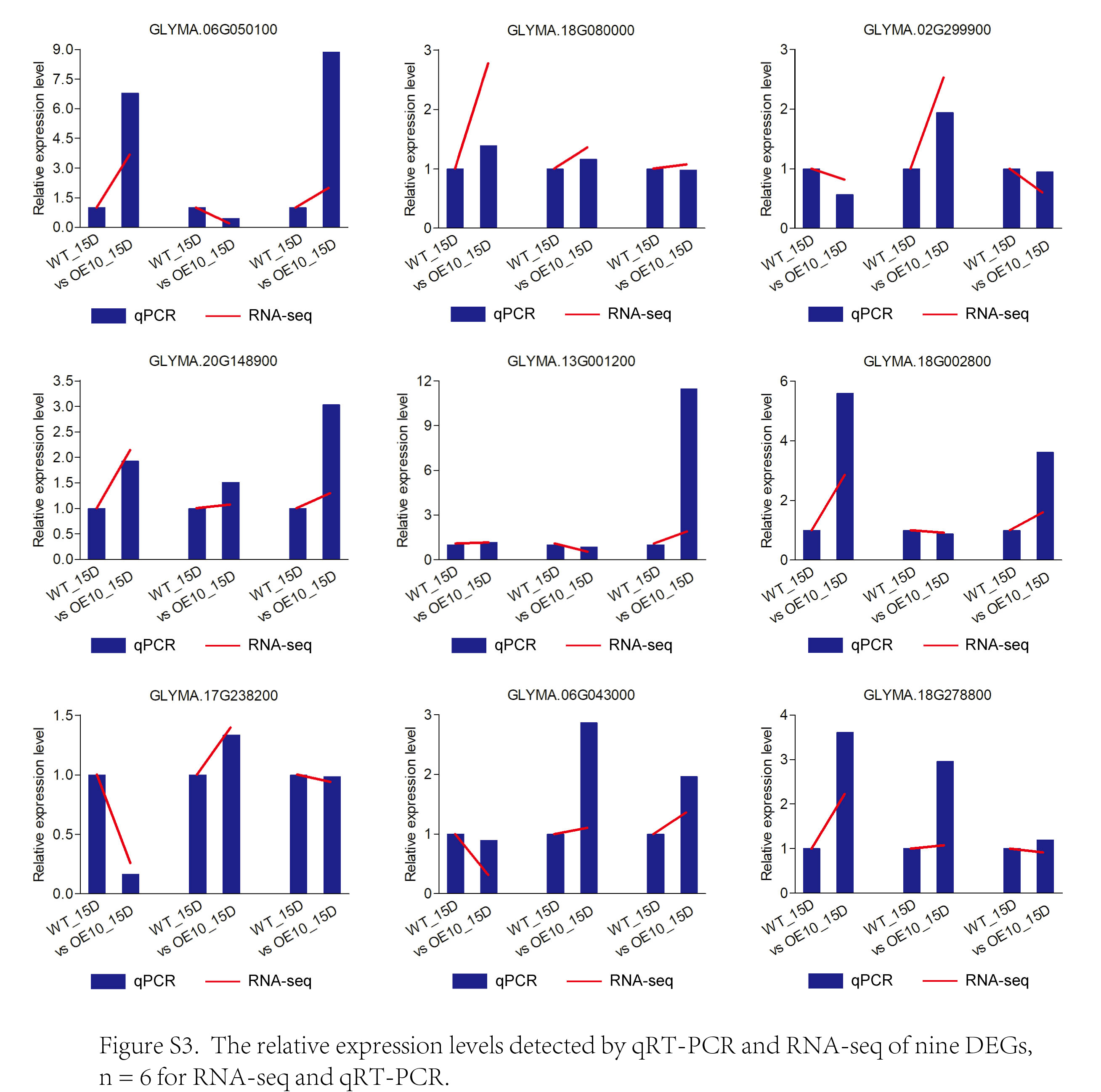

Supplement: Supplementary file 1 [file ijms-24-14125-s001.zip › Figure S3 (2).jpg]
